# Neurite, a Finite Difference Large Scale Parallel Program for the Simulation of Electrical Signal Propagation in Neurites under Mechanical Loading

**DOI:** 10.1371/journal.pone.0116532

**Published:** 2015-02-13

**Authors:** Julián A. García-Grajales, Gabriel Rucabado, Antonio García-Dopico, José-María Peña, Antoine Jérusalem

**Affiliations:** 1 IMDEA Materials Institute, Getafe, Madrid, Spain; 2 DATSI Computer Science, Universidad Politécnica de Madrid, Madrid, Spain; 3 Madrid Supercomputing and Visualization Center; 4 Department of Engineering Science, University of Oxford, Oxford, UK; 5 Mathematical Institute, University of Oxford, Oxford, UK; Hertie Institute for Clinical Brain Research, University of Tuebingen, GERMANY

## Abstract

With the growing body of research on traumatic brain injury and spinal cord injury, computational neuroscience has recently focused its modeling efforts on neuronal functional deficits following mechanical loading. However, in most of these efforts, cell damage is generally only characterized by purely mechanistic criteria, functions of quantities such as stress, strain or their corresponding rates. The modeling of functional deficits in neurites as a consequence of macroscopic mechanical insults has been rarely explored. In particular, a quantitative mechanically based model of electrophysiological impairment in neuronal cells, *Neurite*, has only very recently been proposed. In this paper, we present the implementation details of this model: a finite difference parallel program for simulating electrical signal propagation along neurites under mechanical loading. Following the application of a macroscopic strain at a given strain rate produced by a mechanical insult, *Neurite* is able to simulate the resulting neuronal electrical signal propagation, and thus the corresponding functional deficits. The simulation of the coupled mechanical and electrophysiological behaviors requires computational expensive calculations that increase in complexity as the network of the simulated cells grows. The solvers implemented in *Neurite*—explicit and implicit—were therefore parallelized using graphics processing units in order to reduce the burden of the simulation costs of large scale scenarios. Cable Theory and Hodgkin-Huxley models were implemented to account for the electrophysiological passive and active regions of a neurite, respectively, whereas a coupled mechanical model accounting for the neurite mechanical behavior within its surrounding medium was adopted as a link between electrophysiology and mechanics. This paper provides the details of the parallel implementation of *Neurite*, along with three different application examples: a long myelinated axon, a segmented dendritic tree, and a damaged axon. The capabilities of the program to deal with large scale scenarios, segmented neuronal structures, and functional deficits under mechanical loading are specifically highlighted.

## Introduction

With the recent increase of interest in traumatic brain injuries and spinal cord injuries, a large body of data on their damaging effects is now widely available [1, 2]. However, most of the available research campaigns either focus on the associated cellular level alterations [3–6] or the higher level functional deficits resulting from the associated mechanical insult [7–9]. Only recently, some efforts have been made to link mechanics and electrophysiology in one unique approach [10, 11].

Mainly building on the pioneering work of Hodgkin and Huxley [12], numerous researchers have developed electrophysiological models to simulate the electrical signal propagation in neurons. Fitzhugh, for instance, modeled the saltatory conduction of a myelinated nerve fiber and was able to capture the corresponding action potential (AP) behavior during its propagation [13]. He used the Cable Theory (CT) model [14] for the internodal regions (IRs) and the Hodgkin–Huxley (HH) model for the nodes of Ranvier (NRs). Other authors have explored the relationship between the conduction velocity and the diameter of the fiber. These works identified a linear dependency between both quantities [15, 16], and their relative sensitivity to the nodal area and IR length [17]. Blight and Someya investigated the depolarizing after-potentials: experimentally [18] and with a multi cable model for the myelin sheath [19]. A more recent study focused on the influence of the choice of the myelin sheath model on the electrophysiological properties of the axon [20]. To this end, three different modeling approaches for the myelin electrical behavior were adopted: a perfectly insulating cable [21], a single cable with a finite impedance [13] and a finite impedance double cable model [19]. The first two models exhibited hyperpolarising after-potentials whereas the last model was more accurate with stimulus frequencies above 25 Hz, and produced depolarising after-potentials. McIntyre and coworkers [22] modeled explicitly the NRs, paranodal regions, and IRs with a double cable structure and implemented them in NEURON [23] to study the influence of after-potentials on the recovery cycle of mammalian nerve fibers. Demyelination of axons and associated geometrical effects have also been observed to gradually decrease the conduction velocity until conduction block eventually occurs [24–26]. Following up on such results, drug treatments based on temperature and calcium effects [27] or on the conduction in the damaged region after axonal stretch [28] have been proposed.

More recent modeling efforts have focused on the mechanical aspect of neurons. 3D finite element approaches have been proposed to translate macroscopic strain at the head scale (macroscale) into axonal strain (microscale) for several specific regions in the brain [29]. Other efforts have focused on the blast loading of the cell body of the neuron [30]. In both cases and in other simulation works (see both references for a complete literature review), the functional deficit associated to such mechanical loadings is always left unmodeled. Other models have attempted to account for electrophysiological deficits based on mechanical alterations at the cell level [10, 31]. These models successfully reproduce the observed post axonal blebbing leak of sodium ion channels. These approaches build up on the experimental observation of a “left-shift effect” [32] in the sodium ion current of the portions of the NRs affected by blebbing. Despite such efforts aimed at linking mechanical and geometrical alterations to electrophysiological deficits, a multiscale model relating the macroscopic mechanical loading to functional deficits (i.e., APs propagation) at the tissue scale is still lacking (the previous approaches only modeled the observed left-shift by use of parameters loosely related to damage, but without a direct relation linking one to the other). To explore this problem, the simulator presented in this paper was recently proposed by Jérusalem and coworkers to simulate the electrical signal propagation in Guinea pig spinal cord white matter under mechanical loading [11].

The simulation time for large scale problems becomes naturally longer as the complexity of the simulated neurons grows. Several implementations were thus considered, all of them within the high performance computing discipline: distributed memory multiprocessors (MPI programming) [33, 34], shared memory multiprocessors (OpenMP programming) [35], graphics processing unit (GPU) [36], and many integrated cores (MIC) [37]. GPUs have been chosen due to their high computational power (i.e., several Teraflops when using double precision floating point) and the relative low cost of the middle range GPU cards. Also several GPUs can be used at the same time, in the same host or in different hosts (combining GPUs and MPI), to achieve even higher performance.

In this paper, our in-house program *Neurite* is presented. *Neurite* simulates the electrical signal propagation in myelinated and unmyelinated axons, and in dendritic trees under mechanical loading. As such, *Neurite* is able to simulate the functional deficits in electrical signal propagation with two different solvers (explicit and implicit) and was parallelized using GPUs to reduce the simulation times needed in large scale problems. *Neurite* is a very versatile program that can be adapted to the user’s scenario and can easily be extended with other membrane models for the neurite regions.

## Materials and Methods

The membrane potential is the physical variable that governs the electrical signal propagation along neurites. Both dendrites and axons contribute differently to the electrical behavior of neurons. The electrical signal normally travels from the synaptic inputs to the soma in dendrites, whereas axons transmit the signal from the soma to the axonal tip. Myelinated axons are covered by several insulating layers called myelin sheaths which open up periodically at the NRs, thus giving ion channels access to the extracellular medium [38]. The NRs effectively “boost” the signal during its propagation, shaping the typical saltatory conduction of myelinated axons. IRs are usually modeled as passive regions whereas NRs are modeled by the HH model or some evolutions of this model [10, 13, 14, 19, 28]. Dendrites are usually modeled as passive cables [14, 39].

### Neuronal modeling

**Fig 1:**
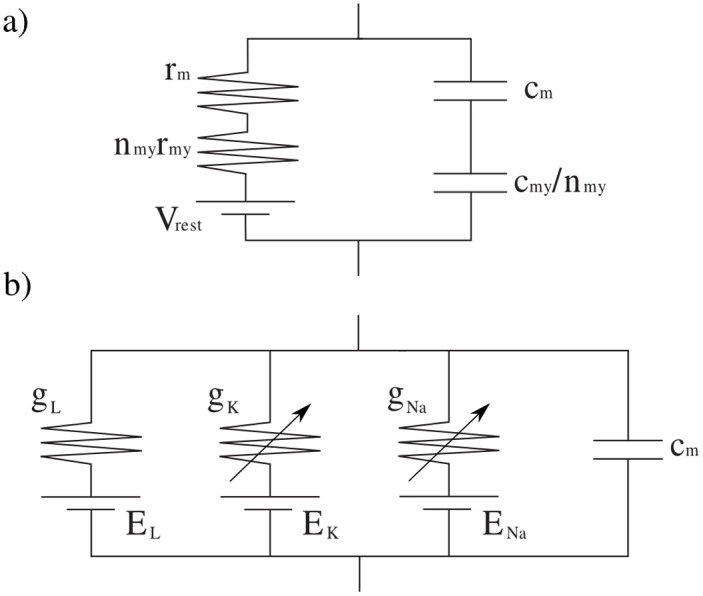
**Membrane models** (*mm*) **available in *Neurite***. a) The CT model is used to simulate all passive regions in the neurites, e.g., the IRs in myelinated axons, b) the HH model is used to simulate all active regions in the neurites, e.g., the NRs in myelinated axons.

*Neurite* models the dendrites and the IRs of myelinated axons as passive cables with the CT model [14]. The NRs and the unmyelinated axons are modeled with the original HH model [12]. The CT equivalent circuit involves the resting membrane potential (*V*_*rest*_), the axial resistivity of the cytoplasm (*ρ*_*a*_), the transmembrane resistivity (*ρ*_*m*_), and the cell membrane electric constant (*C*_*m*_). The presence of myelin layers also involves the consideration of trans-sheath resistivity (*ρ*_*my*_) and electric constant (*C*_*my*_). The HH model adds two new variable conductivities (*G*_*Na*_ and *G*_*k*_) and reversal potentials (*E*_*Na*_ and *E*_*K*_) for the sodium (*Na*_*v*_) and potassium (*K*_*v*_) voltage-gated ion channels considered here. The membrane resistivity is replaced by a leak conductivity (*G*_*L*_) representing the membrane resistivity and other non explicitly modeled channels such as ionic pumps, see Fig. 1.

Both models can be rewritten in a partial differential equation (PDE) form as:

$$A\frac{\partial^{2}V}{\partial x^{2}}=B\frac{\partial V}{\partial t}+CV+D$$ (1)

where *V* is the membrane potential, and *A*, *B*, *C* and *D* parameters are given in Table 1. *E*_*L*_ is the reversal potential associated to the passive leak conductance *G*_*L*_ and is chosen such that *V* = *V*_*rest*_ at rest, i.e.,

$$E_{L}=\left(1+\frac{G_{Na}}{G_{L}}+\frac{G_{K}}{G_{L}}\right)\;V_{rest}-\frac{G_{Na}E_{Na}+G_{K}E_{K}}{G_{L}}$$ (2)

**Table 1:** PDE model parameters.

Parameter	Cable Theory	Hodgkin-Huxley
*A*	$\frac{\pi d^{2}}{4\rho_{a}}$	$\frac{\pi d^{2}}{4\rho_{a}}$
*B*	${\left(\frac{h}{C_{m}\pi d}+\sum_{k=1}^{n_{my}}\frac{h_{my}}{C_{my}\pi d_{my}^{k}}\right)}^{-1}$	$\frac{C_{m}\pi d}{h}$
*C*	$\frac{\pi }{\frac{\rho_{m}h}{d}+\sum_{k=1}^{n_{my}}\frac{\rho_{my}h_{my}}{d_{my}^{k}}}$	$\frac{\pi d}{h}(G_{L}+G_{Na}+G_{K})$
*D*	$\frac{-\pi V_{rest}}{\frac{\rho_{m}h}{d}+\sum_{k=1}^{n_{my}}\frac{\rho_{m}h_{my}}{d_{my}^{k}}}$	$\frac{-\pi d}{h}(G_{L}E_{L}+G_{Na}E_{Na}+G_{K}E_{K})$

*d* and *h* are the neurite diameter and membrane thickness respectively; the subscript *my* indicates that the values are for each one of the *n*_*my*_ myelin layers.

Note that this value of *E*_*L*_ remains constant throughout the simulation under the assumption that the ion homeostasis exchangers would not be damaged during deformation, but would try to accommodate the changes in concentrations due to alterations of *Na*_*v*_ and *K*_*v*_, see Ref. [11] for more details.

For the particular equations of the HH model, the conductances are variable and depend on the current potential *V* and on two constants ${\bar{G}}_{Na}$ and ${\bar{G}}_{K}$ corresponding to the channel conductivities when fully open [12]. The evolution equations for *G*_*Na*_ and *G*_*K*_ used by *Neurite* are shown in Table 2. In this table, the dimensionless activation (*m* and *n*) and inactivation (*h*) particles describe the evolution of the corresponding conductances as a function of the rate constants *α*_*k*_ and *β*_*k*_ for *k* ∈ {*m*, *h*, *n*}.

**Table 2:** Hodgkin-Huxley parameters.

*Na*_*v*_	*K*_*v*_
$G_{Na}(V)={\bar{G}}_{Na}m^{3}h$	$G_{K}(V)={\bar{G}}_{K}n^{4}$
$\frac{dm}{dt}=\alpha_{m}(V)(1-m)+\beta_{m}(V)m$	$\frac{dn}{dt}=\alpha_{n}(V)(1-n)+\beta_{n}(V)n$
$\frac{dh}{dt}=\alpha_{h}(V)(1-h)+\beta_{h}(V)h$	
$\alpha_{m}(V)=\frac{25-(V-V_{rest})}{10\;\left(e^{\frac{25-(V-V_{rest})}{10}}-1\right)}$	$\alpha_{n}(V)=\frac{10-(V-V_{rest})}{100\;\left(e^{\frac{10-(V-V_{rest})}{10}}-1\right)}$
$\alpha_{h}(V)=0.007e^{\frac{-(V-V_{rest})}{20}}$	
$\beta_{m}(V)=4e^{\frac{-(V-V_{rest})}{18}}$	$\beta_{n}(V)=0.125e^{\frac{-(V-V_{rest})}{80}}$
$\beta_{h}(V)=\frac{1}{e^{\frac{30-(V-V_{rest})}{10}}+1}$	

*Na*_*v*_ and *K*_*v*_ parameters. Potential and time units are, respectively, *mV* and *ms* in this table. Note that ${\bar{G}}_{Na}$ and ${\bar{G}}_{K}$ are the maximal *Na*_*v*_ and *K*_*v*_ conductances, respectively, and are taken from the original HH model [12].

### Spatial discretization

**Fig 2:**
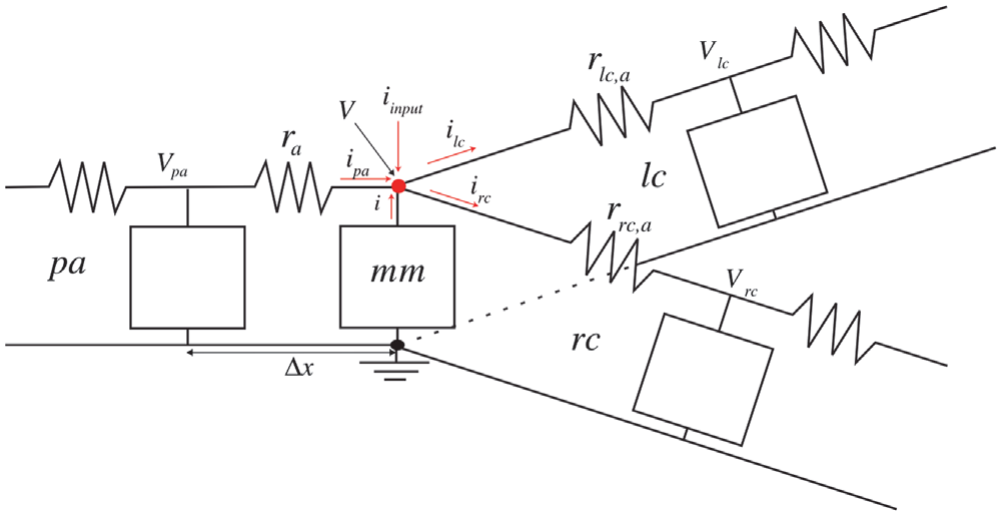
General discretization framework. Each element *i* (and its corresponding *mm*) is related to its *pa*, *rc*, and *lc* in the case that *i* is at a branching point (if not, *lc* does not exist).

*Neurite* solves Equation (1) using the finite difference method (FDM) originally developed by A. Thom in the 1920s to solve non-linear hydrodynamics equations [40]. The PDE is discretized in time (subsequently, *n* subscript) and space. Each increment of time is done by a time step Δ*t*, whereas each increment in space is an element with the following characteristics: its membrane model *mm*, corresponding to either CT or HH; its element size Δ*x*; its parent element *pa*; its right child element *rc*; a possible left child element *lc*; and finally a flag *fb* indicating if the element is at a branching point, see Fig. 2. Note that, although the “right” and “left” terms are arbitrary, in this work “right” denotes the first branch and “left” the second one (which only exists at a branching point).

Applying the first Kirchhoff law to the general case (i.e., with *lc*), the equilibrium reads:

$$i_{lc}\;\text{+}\;i_{rc}\;\text{−}\;i\;\text{−}\;i_{pa}\;\text{−}\;i_{input}=0$$ (3)

where *i*_*lc*_ and *i*_*rc*_ are the currents flowing through the corresponding children, *i* the current passing through the membrane and potential myelin layers (two possibilities so far: CT or HH model), *i*_*pa*_ the current coming from the parent, and finally *i*_*input*_ a possible external current (to mimic the input signal at any point of the neurite). Note that *i*_*lc*_ is zero (and *fb* is *false*) when the element is not at a branching point.

The currents are related to their corresponding potentials *V*_*α*_ where *α* ∈ {∅, *pa*, *rc*, *lc*} (see Fig. 2) as follows:

{ilc=V−Vlcrlc,airc=V−Vrcrrc,aipa=Vpa−Vrai=cmmdVdt+WV+K} (4)

where

$$r_{\alpha ,a}=\underset{{\hat{r}}_{\alpha ,a}}{\underset{︸}{\frac{4\rho_{\alpha ,a}}{\pi d_{\alpha }^{2}}}}\Delta x_{\alpha }$$ (5)

For the membrane (myelinated for IRs) current in Equation (4) the capacitance reads:

$$c_{mm}=\underset{{\hat{c}}_{m}}{\underset{︸}{{\left(\frac{h}{C_{m}\pi d}+\sum_{k=1}^{n_{my}}\frac{h_{my}}{C_{my}\pi d_{my}^{k}}\right)}^{-1}}}\Delta x$$ (6)

where the number of myelin layers *n*_*my*_ wrapping the IRs is set to zero (i.e., the second term of the equation is discarded) for NRs or passive dendritic tree (barring a few exceptions [41, 42], dendritic trees are unmyelinated), and $d_{my}^{k}=d+2h+2(k-1)h_{my}$, where *d*, *h* and *h*_*my*_ are the neurite diameter, and the membrane and myelin layer thicknesses, respectively.

*W* and *K* are parameters that depend on the kind of model used; if the element is a CT element the values are constant:

{W=−1rmK=Vrestrm} (7)

where *r*_*m*_ is given by:

$$r_{m}=\underset{{\hat{r}}_{m}}{\underset{︸}{\left(\frac{\rho_{m}h}{\pi d}+\sum_{k=1}^{n_{my}}\frac{\rho_{my}h_{my}}{\pi d_{my}^{k}}\right)}}\frac{1}{\Delta x}$$ (8)

whereas if the element is a HH element, then *W* and *K* are functions of several conductances that depend on the potential and time:

{W=−(gNa(V)+gK(V)+gL)K=gNa(V)ENa+gK(V)EK+gLEL} (9)

where

{gNa(V)=πdGNa(V)h︸g^NaΔxgK(V)=πdGK(V)h︸g^KΔxgL=πdGLh︸g^LΔx} (10)

### Mechanical alterations

**Fig 3:**
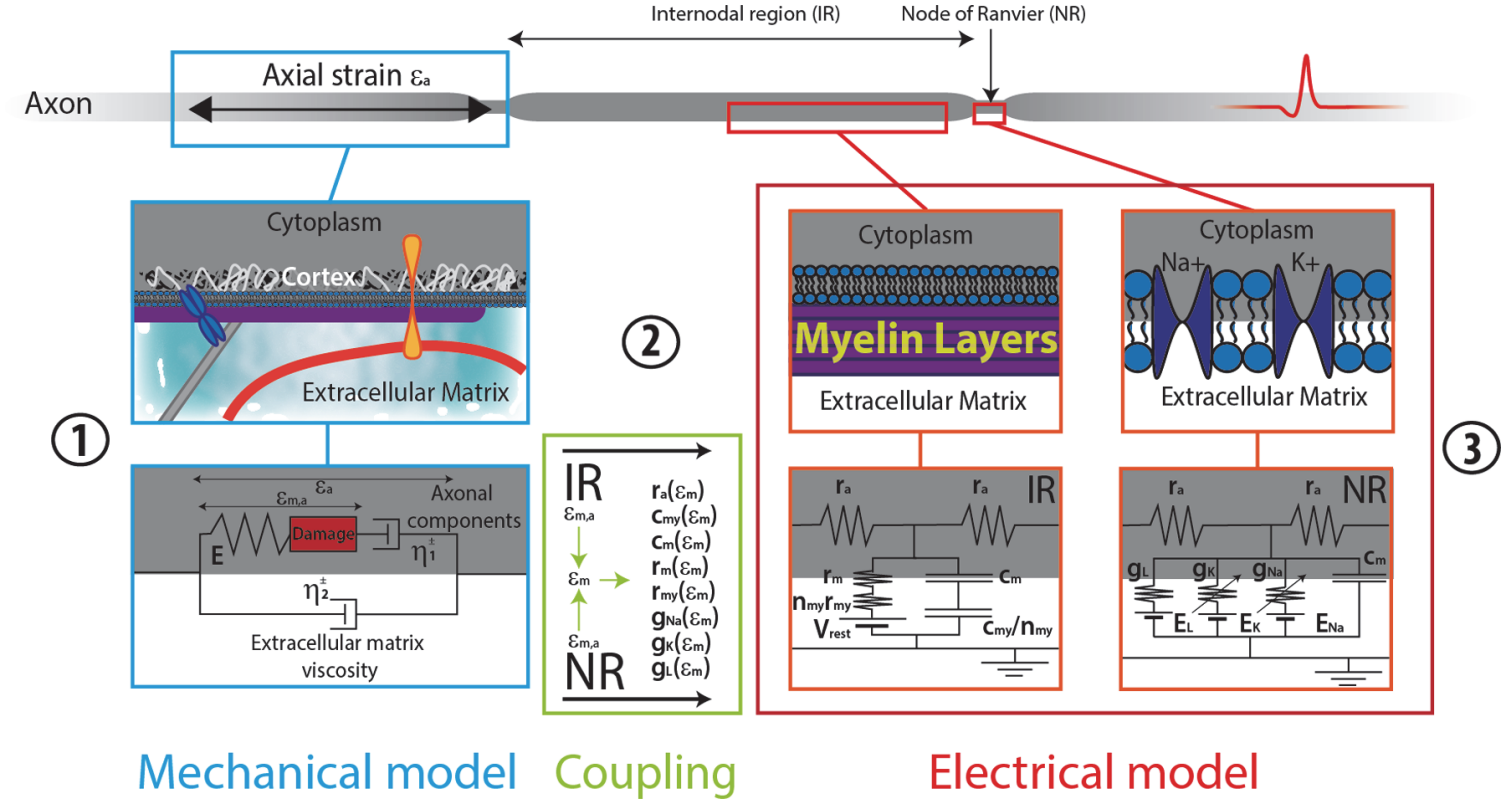
Full model. The mechanical model **①** transforms the macroscopic strain and strain rate into their microscopic counterparts, which is then used by the coupling model **②** to modify the parameters of the electrophysiological model **③** to eventually quantify the functional deficits in the electrical signal propagation (this picture has been reproduced with permission of the authors and the journal of Ref. [11]).

All equations exposed until here are purely electrophysiological in nature and do not account explicitly for any alteration produced by a mechanical insult. The full model is shown in Fig. 3 for the specific case of an axon. The mechanical model is composed of several components that represent the neurite mechano-electrophysiological behavior under a mechanical loading characterized by a macroscopic strain at a corresponding strain rate. The main features of the model are summarized in the following, see Ref. [11] for more details.

The microscopic electrophysiological alterations produced by the macroscopic strain and strain rate of the mechanical model are directly leading to geometrical modifications in the diameter *d* and the size Δ*x* for each element:

{d=d01+ϵm,aΔx=Δx0(1+ϵm,a)} (11)

where *ε*_*m*,*a*_ is the microscopic axial neurite strain, and where *d*_0_ and Δ*x*_0_ are their respective reference values (no strain).

These purely geometrical alterations are coupled to a damage based criterion directly affecting the health of the ion channels. This alteration mechanism accounts for the *Na*_*v*_ current “left-shift” experimentally observed in the stretch-induced alterations of the membrane [32]. The reversal potentials and probabilities of the channels are thus modified accordingly [11]. For more details, see Equations (9–10) of Ref. [11] for the intrinsic mechanisms of this damage based alteration. A complete discussion on the choice of using similar mechanistic alterations for *K*_*v*_ is also provided.

To summarize, a mechanical loading at a given strain and strain rate is given as an input of the mechanical model (Fig. 3-①), a microscopic strain directly affecting the membrane components is deduced and used to modify the electrophysiological model parameters (Fig. 3-②), and ultimately, the electrophysiological model is used to study the resulting signal propagation (Fig. 3-③).

### Explicit scheme

The explicit scheme uses forward difference in time for the first order derivative and second order central difference for the spatial derivative. This scheme relates each variable at time *n*+1 to the same variable and its neighbors’ at time *n*. Its application to Equation (4) for all elements leads to

$$\frac{{\hat{c}}_{m}\Delta x}{\Delta t}\left(V^{n+1}-V^{n}\right)-W\Delta xV^{n}-K\Delta x+\frac{V^{n}-V_{pa}^{n}}{{\hat{r}}_{a}\Delta x}+fb\frac{V^{n}-V_{lc}^{n}}{{\hat{r}}_{lc,a}\Delta x_{lc}}+\frac{V^{n}-V_{rc}^{n}}{{\hat{r}}_{rc,a}\Delta x_{rc}}-i_{input}=0$$ (12)

and finally

$$V^{n+1}=V^{n}+\frac{\Delta t}{{\hat{c}}_{m}\Delta x}(W\Delta xV^{n}+K\Delta x+\frac{V_{pa}^{n}-V^{n}}{{\hat{r}}_{a}\Delta x}+fb\frac{V_{lc}^{n}-V^{n}}{{\hat{r}}_{lc,a}\Delta x_{lc}}+\frac{V_{rc}^{n}-V^{n}}{{\hat{r}}_{rc,a}\Delta x_{rc}}+i_{input})$$ (13)

### Implicit scheme

The implicit scheme method uses backward difference in time and second order central difference for the spatial derivative. The current state of each element is calculated in function of its previous state and of the current state of its neighbors. Applying this scheme to Equation (4) leads to

$$\frac{{\hat{c}}_{m}\Delta x}{\Delta t}\left(V^{n+1}-V^{n}\right)-W\Delta xV^{n+1}-K\Delta x+\frac{V^{n+1}-V_{pa}^{n+1}}{{\hat{r}}_{a}\Delta x}+fb\frac{V^{n+1}-V_{lc}^{n+1}}{{\hat{r}}_{lc,a}\Delta x_{lc}}+\frac{V^{n+1}-V_{rc}^{n+1}}{{\hat{r}}_{rc,a}\Delta x_{rc}}-i_{input}=0$$ (14)

which can be rewritten as

$$\alpha V_{pa}^{n+1}+\beta V^{n+1}+\gamma V_{lc}^{n+1}+\delta V_{rc}^{n+1}=b$$ (15)

where

{α=−1r^aΔxβ=c^mΔxΔt−WΔx+fgr^lc,aΔxlc+1r^rc,aΔxrc+1r^aΔxγ=−fgr^lc,aΔxlcδ=−1r^rc,aΔxrcb(Vn)=c^mΔxΔtVn+KΔx+iinput} (16)

This, in turn, can be rewritten in matrix form as

$$\begin{array}{cc} & \;\;\;\;\;\;\;\;\;\;\;\;\;\;\;\;\underset{↓}{k}\;\;\;\;\;\;\;\;\;\;\;\underset{↓}{l}\\ & \begin{array}{c} k\to \\ l\to \end{array}\underset{\tilde{A}}{\underset{︸}{\left(\begin{array}{ccccccccccccccc} \beta & \delta & 0 & \ldots & \ldots & \ldots & \ldots & \ldots & \ldots & \ldots & \ldots & \ldots & \ldots & \ldots & 0\\ \alpha & \beta & \delta & 0 & \ldots & \ldots & \ldots & \ldots & \ldots & \ldots & \ldots & \ldots & \ldots & \ldots & 0\\ 0 & \ddots & \ddots & \ddots & & & & & & & & & & & \vdots \\ \vdots & & \ddots & \ddots & \ddots & & & & & & & & & & \vdots \\ 0 & \ldots & 0 & \alpha & \beta & \delta & 0 & \ldots & 0 & \gamma & 0 & \ldots & \ldots & \ldots & 0\\ 0 & \ldots & \ldots & 0 & \alpha & \beta & \delta & 0 & \ldots & \ldots & \ldots & \ldots & \ldots & \ldots & 0\\ \vdots & & & & & \ddots & \ddots & \ddots & & & & & & & \vdots \\ 0 & \ldots & \ldots & \ldots & \ldots & 0 & \alpha & \beta & \delta & 0 & \ldots & \ldots & \ldots & \ldots & 0\\ 0 & \ldots & \ldots & \ldots & \ldots & \ldots & 0 & \alpha & \beta & \delta & 0 & \ldots & \ldots & \ldots & 0\\ 0 & \ldots & \ldots & 0 & \alpha & 0 & \ldots & \ldots & 0 & \beta & \delta & 0 & \ldots & \ldots & 0\\ 0 & \ldots & \ldots & \ldots & \ldots & \ldots & \ldots & \ldots & 0 & \alpha & \beta & \delta & 0 & \ldots & 0\\ \vdots & & & & & & & & & & \ddots & \ddots & \ddots & & \vdots \\ 0 & \ldots & \ldots & \ldots & \ldots & \ldots & \ldots & \ldots & \ldots & \ldots & 0 & \alpha & \beta & \delta & 0\\ 0 & \ldots & \ldots & \ldots & \ldots & \ldots & \ldots & \ldots & \ldots & \ldots & \ldots & 0 & \alpha & \beta & \delta \\ & \ldots & \ldots & \ldots & \ldots & \ldots & \ldots & \ldots & \ldots & \ldots & \ldots & \ldots & 0 & \alpha & \beta \end{array}\right)}}\cdot \;V^{n+1}=b \end{array}$$ (17)

where the bold forms **V** ^*n*+1^ and **b** of *V* ^*n*+1^ and *b* are the vectors of the corresponding values for all elements. In this example, one branching between elements *k*, *k*+1, and *l* can be identified by the presence of *γ* at row *k*, column *l*, and an off-tridiagonal *α* at row *l*, column *k*.

### Boundary conditions

The general boundary condition applied to the terminal elements is a *sealed-end boundary condition* [14]:

$$\frac{\partial V}{\partial x}=0$$ (18)

For the first element, this is reinforced by equalling its potential to the following one (*V*_0_ = *V*_1_) and a branching point is thus not allowed at the first element. For the remaining terminal elements, the potential is equalled to the potential of its parent *V*_*terminal*_ = *V*_*pa*_.

In the explicit scheme, the boundary condition is directly applied at each time step. In the implicit scheme, $\tilde{A}$ of Equation (17) is modified as follows

$$\tilde{\text{A}}=\left(\begin{array}{ccccccccccccccc} 1 & 0 & 0 & \ldots & \ldots & \ldots & \ldots & \ldots & \ldots & \ldots & \ldots & \ldots & \ldots & \ldots & 0\\ 0 & \alpha +\beta & \delta & 0 & \ldots & \ldots & \ldots & \ldots & \ldots & \ldots & \ldots & \ldots & \ldots & \ldots & 0\\ 0 & \ddots & \ddots & \ddots & & & & & & & & & & & \vdots \\ \vdots & & \ddots & \ddots & \ddots & & & & & & & & & & \vdots \\ 0 & \ldots & 0 & \alpha & \beta & \delta & 0 & \ldots & 0 & \gamma & 0 & \ldots & \ldots & \ldots & 0\\ 0 & \ldots & \ldots & 0 & \alpha & \beta & \delta & 0 & \ldots & \ldots & \ldots & \ldots & \ldots & \ldots & 0\\ \vdots & & & & & \ddots & \ddots & \ddots & & & & & & & \vdots \\ 0 & \ldots & \ldots & \ldots & \ldots & 0 & \alpha & \beta +\delta & 0 & 0 & \ldots & \ldots & \ldots & \ldots & 0\\ 0 & \ldots & \ldots & \ldots & \ldots & \ldots & 0 & 0 & 1 & 0 & 0 & \ldots & \ldots & \ldots & 0\\ 0 & \ldots & \ldots & 0 & \alpha & 0 & \ldots & \ldots & 0 & \beta & \delta & 0 & \ldots & \ldots & 0\\ 0 & \ldots & \ldots & \ldots & \ldots & \ldots & \ldots & \ldots & 0 & \alpha & \beta & \delta & 0 & \ldots & 0\\ \vdots & & & & & & & & & & \ddots & \ddots & \ddots & & \vdots \\ 0 & \ldots & \ldots & \ldots & \ldots & \ldots & \ldots & \ldots & \ldots & \ldots & 0 & \alpha & \beta & \delta & 0\\ 0 & \ldots & \ldots & \ldots & \ldots & \ldots & \ldots & \ldots & \ldots & \ldots & \ldots & 0 & \alpha & \beta +\delta & 0\\ & \ldots & \ldots & \ldots & \ldots & \ldots & \ldots & \ldots & \ldots & \ldots & \ldots & \ldots & 0 & 0 & 1 \end{array}\right)$$ (19)

### Numbering scheme

The proposed enumeration is motivated by some solver requirements in the program such as the management of the terminal elements, as well as the possibility to represent the system with a quasi-tridiagonal matrix, see Equation (19). Following Fig. 2, all elements have a *pa* and an *rc*. If the element is at a branching point, then it also has an *lc*, and *fg* is *true*. When the element is a terminal element its *rc* is taken as itself. The following guidelines were adopted to simplify the construction of the matrices and vectors needed by the solvers.

**Fig 4:**
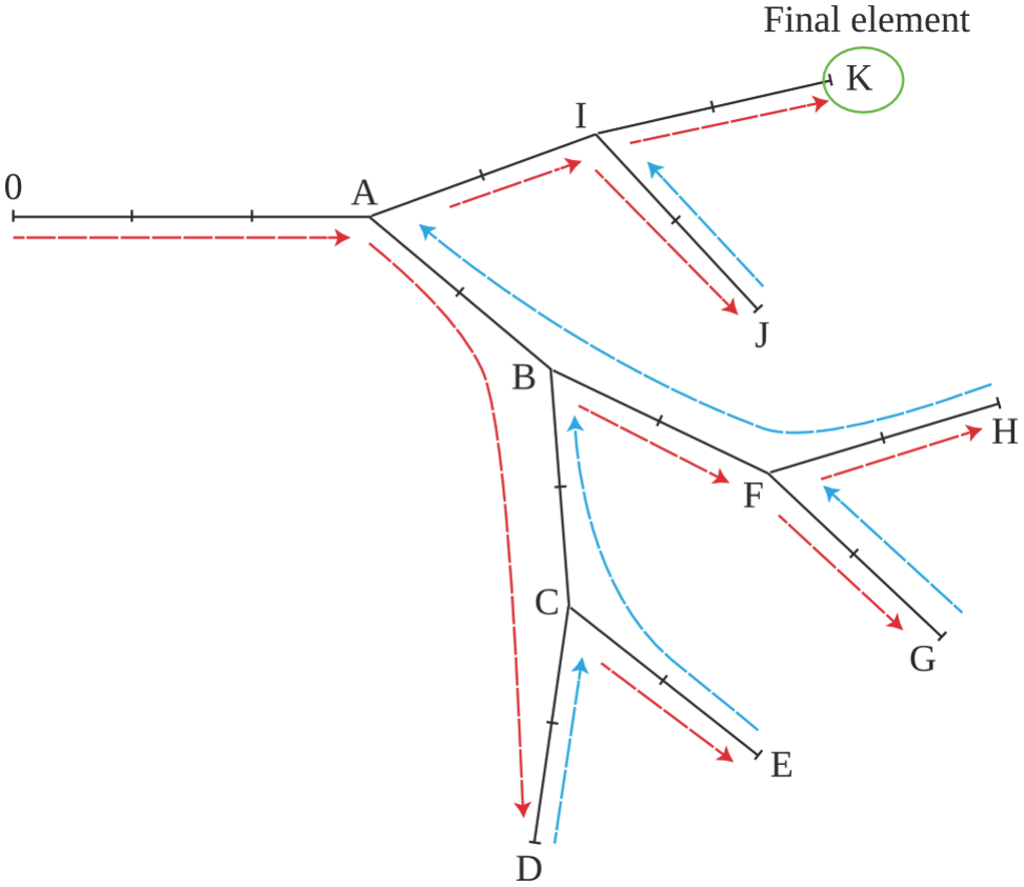
General tree enumeration. The tree begins at element 0 and continues enumerating the elements following the red arrows. When a terminal element is reached, the enumeration returns to the immediate previous unfinished branch.

The enumeration always goes from the soma to the neurite tips. When a branching point is reached, it continues through the right branch. When the enumeration reaches a terminal element, it comes back to the immediate previous unfinished branching and continues with the same rules until the final element is reached. In the example given in Fig. 4, the enumeration begins at 0 and goes until *A*, it continues through *C* until it reaches *D*. Since *D* is a terminal element, the enumeration comes back to the previous branching point, and continues from *C* to *E*. Following the same rules, the enumeration will finally walk on the following path: *B* → *F* → *G* → *F* → *H* → *A* → *I* → *J* → *I* → *K*. Note that this graph implies that the 3D structure needs to be “flattened” before being used by *Neurite*.

### *Neurite* convergence

Following the *Lax equivalence theorem*, the FDM schemes used here are convergent as long as consistency and stability are verified [43]. The validation of *Neurite* against the Rallpacks benchmarks [44] shows satisfactory results for all applications suggested by Rallpacks, with a suitable accuracy and simulation speeds when compared to the analytical solutions (when they exist), and to NEURON and GENESIS (when the analytical solutions do not exist), see S1 File for a description of the validation procedure.

A FDM is said consistent if the solution calculated at a given coordinate (or given time) converges to its analytical PDE solution when Δ*x* (or Δ*t*) goes to zero. The consistency of both schemes was systematically validated for both discretizations.

In order to study the temporal stability of the explicit scheme, a spectral analysis was done. To this end, the constant terms in the Equation (1) are neglected and Equation (12) can be rewritten as:

$$\hat{A}\cdot V^{n}=V^{n+1}$$ (20)

where $\hat{A}$ is a sparse matrix. In order to simplify the identification of its spectral radius *ρ* (the largest absolute value of the eigenvalues), the branching pattern is neglected (*lc* = 0 and *fb* = *false* for all elements). Ignoring the boundary conditions, $\hat{A}$ is thus tridiagonal. The stability condition *ρ* < 1 is equivalent to:

{Δt<ΔtcΔtc=min(2BC+AΔxi2),i∈{1,2,…,N}} (21)

where *A*, *B* and *C* are given in Table 1, and where *N* is the total number of elements in the spatial discretization. Note that depending on the spatial discretization defined by the user (Δ*x*), the computed Δ*t* can thus vary within a wide range (see S1 File for more details). Since the spectral analysis for the implicit scheme is considerably more complex, the temporal implicit stability was only empirically observed for a larger range of Δ*t*, as expected.

A similar spectral analysis was done for the central difference spatial discretization (second order derivative). To this end, the time was assumed continuous and only the space was discretized. Taking the same assumptions as above (neglecting the branching pattern, the constant terms, and the boundary conditions), the resultant structure reads

$$\bar{A}\cdot V=\frac{\partial V}{\partial t}$$ (22)

where $\bar{A}$ is a tridiagonal matrix.

The study of the spectral radius of $\bar{A}$ leads to an unconditional spatial stability.

Albeit defined here for very special cases, these stability rules were shown to be respected for all the configurations studied in this work.

### Implementation

*Neurite* has been implemented in C++. Each solver is clearly differentiated with its own C++ prototype (with its corresponding header file). The scenario configuration must provide a set of arrays with the spatial discretization, define the stimulus currents, set the total time of the simulation, and define the outputs of the simulation. *Neurite* then calculates Δ*t*_*c*_ for the explicit scheme and the simulation is run with Δ*t* = *η*Δ*t*_*c*_ (*η* is the scale factor), with *η* ≫ 1 for the implicit scheme and *η* ≤ 1 for the explicit scheme.

So as to define the spatial discretization, the scenario setup must provide a creating/loading function depending on whether the neurite is synthetically defined or loaded from the geometry of a neuron segmented from experimental data. Several functions in *Neurite* have been implemented to create typical spatial discretizations such as myelinated axons or random symmetric dendritic trees. When *Neurite* is loading a segmented neuron, this neuron must be adapted to *Neurite*’s enumeration.

The explicit and implicit solvers are implemented for CPUs for simple problems with a reasonable number of elements and for GPUs to obtain faster parallel simulations with an extremely large amount of elements (thus allowing *Neurite* to simulate full neurons or in the near future, small networks). The mathematical simplicity of the FDM allows a straight and easy parallel implementation of both solvers. In view of the expected growth in complexity of the simulated scenarios (e.g., whole neurons, small networks, damaged neurites, etc.), it thus a priori presents a definite advantage on other approaches. Whereas the explicit approach might be more time consuming than the implicit method, its robustness is also guaranteed for even complex non-linear constitutive models. As a consequence, both approaches are presented here.

#### CPU and GPU solvers for the explicit scheme

In this scheme, the program consists of three main phases: (i) initialization, where all the variables are created and values are assigned; (ii) updating, where the variables used in the calculation are updated; and (iii) calculation, where the membrane potential is computed. The first phase is performed once at the beginning of the execution, while the other two phases (updating and calculation) are performed at each time step. A profiling study shows that the execution time is mainly consumed equally by the updating and the calculation phases.

As discussed earlier, a neurite can have two types of elements, HH or CT. Furthermore, each of these elements can be a branching, terminal or normal element. In the CPU solver, these elements have been implemented in C++ using an “element” class, with common properties and methods. From this class, two subclasses (or possible specializations) are derived: HH and CT with individual properties that are not shared by both types of elements.

In the GPU version, the element vector is split into several vectors to improve the performance. A GPU works as a vector processor executing the same instruction over different sets of data at the same time. Thus, several of them must be read at the same time to avoid waiting for the data to be processed. Once the instruction is executed, the results must also be written at the same time. In order to improve the performance of the memory hierarchy, the code must have unit-stride data accesses, which is done by having contiguous data in the memory. It is thus better to have a structure of vectors than a vector of structures since the spatial proximity of references is improved, as all the data of the same type are contiguous and they can be read or written at the same time. Applying this technique to the element vector, four vectors (possible intersecting) are obtained: the terminal elements, the branch elements, the HH elements and the global vector containing all the elements (including the previous three vectors and the CT elements).

The updating and calculation functions are specialized. In the CPU implementation, flags are used to treat each element type: terminal, branch or normal, and HH or CT. These flags generate bifurcations that are inefficient for the GPUs. The calculation function was thus divided into three functions, each one focused on calculating a particular element:

- *Terminal*: Used with the vector of terminal elements
- *Branch*: Used with the vector of branch elements
- *Normal*: The other elements

Each function thus only performs the necessary operations on a single vector that has only one type of elements. The number of terminal and branch elements is negligible compared to the number of normal elements. Consequently, the *Normal* calculation function goes over all the elements, including those that are of branch and terminal type, and then the *Branch* and *Terminal* functions are executed and overwrite the previous values. This redundant computation is inexpensive as few elements are recalculated.

A fourth function updates the HH element vector. In this case, the code of the called functions were included to avoid nested calls and simplify the transport to the GPU.

These four functions must perform the same steps as in the CPU version, except that instead of reserving memory on the host they must reserve memory on the GPU. As a consequence, the memory used by the simulation resides in the GPU and memory transfers between the host and the GPU in the middle of the simulation are avoided. Each of these four functions was implemented as a kernel. Finally, two GPU streams were created in order to execute in parallel the computation of the terminal and branch elements, since the elements of these two types are independent and can be computed at the same time.

#### CPU and GPU solvers for the implicit scheme

In this scheme, a linear system of equations needs to be solved. The resulting matrix $\tilde{A}$ is a sparse matrix, and can be stored in a 3-array variation of the compressed sparse row format. A flexible generalized minimal residual method (FGMRES), provided by the Math Kernel Library (MKL) of Intel [45], solves the linear system at each time step in the CPU sequential version and a biconjugate gradient stabilized method (BICGSTAB) was implemented to solve the linear system in the GPU parallel version.

For the GPU version, available libraries such as *Paralution* [46] or *Cusp* [47] were found to have major limitations in performance due to the generated memory transfers between CPU and GPU. At each time step arrays and vectors need to be updated and transferred from the host to the GPU, the system of equations needs to be solved using the library, and the results finally transferred from the GPU to the host. To avoid this traffic, an algorithm creates the matrices and transfer them to the GPUs, perform all the computations on the GPUs and transfer back the results only. Additionally, the transfer of the results of an iteration is done in parallel with the computation of the next iteration.

The update function does the same tasks as the one of the explicit scheme, but also updates the overall matrices and right hand side vectors of the implicit scheme. For each of these tasks a separate kernel was implemented, since matrices and vectors can be updated in parallel. Performing the update on the GPU removes the problem of the memory transfers. The systems are solved with BICGSTAB, a robust and fast numerical method relying on the mathematical library provided by Nvidia CUBLAS [48].

## Results and discussion

In order to evaluate the capabilities of *Neurite*, this paper presents a series of mid-complexity scenarios. *Neurite* is able to simulate the electrical signal propagation in a dendritic tree or in myelinated or unmyelinated axons. This first version of the program is very adaptable to other models of ion channels, electrical passive models, or myelin layers. To illustrate this flexibility, we present three different applications: (i) a long myelinated axon with a considerable number of elements, aimed at showing the benefits of the parallel implementation of the solvers; (ii) a segmented dendritic branch obtained from the *NeuroMorpho.Org* [49] database; and (iii) a stretched myelinated axon to evaluate the functional deficits in the AP propagation by use of the mechanical model included in *Neurite* and proposed by Jérusalem *et al*. [11]. All solvers and implementations are used and compared for the three application examples.

### Myelinated axon

The myelinated axon is composed of two different regions (IR and NR). A very long axon (*L* ∼ 2*m*) with a considerable number of elements is used (such axons can actually be found in giraffes [50]) to study (and leverage) the benefits of the parallel implementations of the solvers. Tracking the AP propagation along the whole length of the axon leads to a considerable number of time steps (see Table 3). In this scenario, the performances of both solvers with both type of processors are compared. The mechanical model is disregarded in this example (i.e., the electrophysiological properties are not altered by any deformation). The set of parameters for these simulations are taken from the literature [11, 14].

**Table 3:** Time consumptions for the myelinated axon.

Number of time steps	Solver	CPU (s)	GPU (s)	Speedup
7,661,965	Explicit	76,129	1,476	51
45,971	Implicit	278,942	3,806	71

The total number of elements is 251,894.

The total number of elements (∼ 251,000) is distributed in CT and HH elements. The critical time step is calculated to be Δ*t*_*c*_ = 13 *ns* and the time step is Δ*t* = *η*Δ*t*_*c*_ with *η* = 0.6 and *η* = 100 for the explicit and implicit schemes, respectively. The objective of this application is only to show the performance of the solvers and the advantages of the parallel implementations. The execution times are shown in Table 3.

### Segmented passive dendritic tree

**Fig 5:**
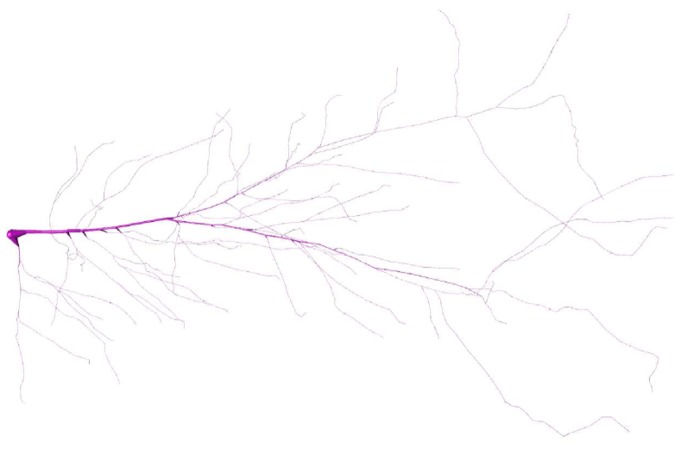
Segmented dendritic tree [51]. This adapted version is visualized with Vaa3D [63]. The tree consists of 57 branching points and 879 elements.

*Neurite* is able to load segmented neuronal geometries, with only few adaptations. For this example, a segmented structure was taken from the *NeuroMorpho.Org* database [49]. In this repository, segmented neurons including the dendrites, apical dendrite, soma and axon can be downloaded. For this example, a pyramidal neuron of rat hippocampus was chosen (*NeuroMorpho.org* ID: *NMO*_00223, [51]), and the simulation was reduced to the dendritic tree using CT passive elements with arbitrary properties, see Fig. 5 (the soma is shown for illustration but was not included in the simulation).

The tree has 57 branching points and 879 elements. The critical time step is Δ*t*_*c*_ = 62 *ns* and the time step is Δ*t* = *η*Δ*t*_*c*_, with *η* = 0.6 and *η* = 100 for the explicit and implicit schemes, respectively. The execution times are shown in Table 4.

**Table 4:** Time consumptions for the dendritic tree.

Number of time steps	Solver	CPU (s)	GPU(s)	Speedup
21,381,287	Explicit	816	544	1.5
96,218	Implicit	280	1,800	0.16

The total number of elements is 879.

### Damaged axon

In this example, *Neurite* is used to quantify the functional deficits in the AP propagation of an axon under mechanical loading. The full study of the mechanical model and its implementation in *Neurite* have been published for spinal cord Guinea pig white matter [11]. These results were validated against experimental results published in Ref. [7]. In the example taken here, the AP decreases at the measurement point, for a mild axial macroscopic strain (25%) at fast axial strain rate (∼ 400 *s* ^−1^). See Ref. [11] for more details.

**Fig 6:**
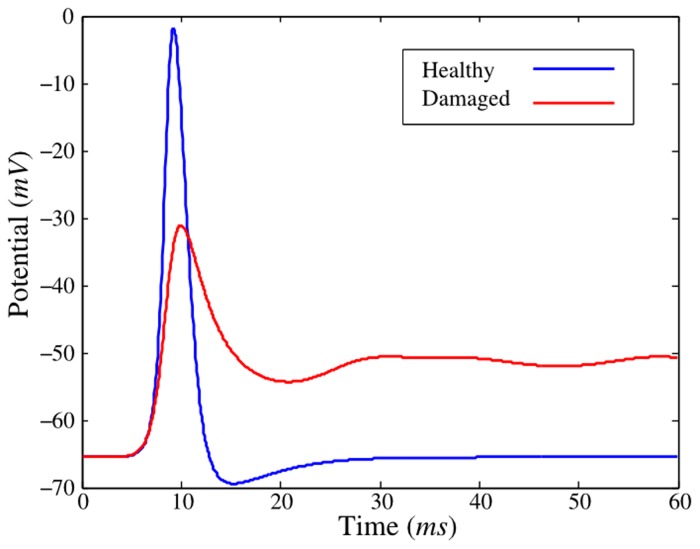
APs propagation for healthy and damaged axons. The decrease in the potential corresponds to a mild axial macroscopic strain (25%) at fast axial strain rate (∼ 400 *s* ^−1^), see Ref. [11] for more details.

The results exposed in Fig. 6 show the potential at a given point for both damaged and healthy axons. The unstretched axon is 10 *mm* in length and all parameters used in this example are the same as in Ref. [11]. This multiscale approach is a novelty in the field, linking the electrophysiological properties of the membrane at the NRs and IRs to the deformation and damage of the whole axon. In the full original study, the CPU explicit solver was used and *Neurite* was executed many times for the calibration (∼ 5,000 simulations). An additional implicit calculation with *η* = 100 was done here. The execution times are shown in Table 5.

**Table 5:** Time consumptions for the damaged axon.

Number of time steps	Solver	CPU (s)	GPU(s)	Speedup
1,402,302	Explicit	30	25	1.21
467,434	Implicit	10	81	0.13

The total number of elements is 525.

### Comparison of the solvers and processors

Both solvers (explicit and implicit) with both processors (CPU and GPU) were used for all application examples shown in this paper. A summary of the configuration and the results for all simulations are exposed in Tables 3, 4 and 5. All the measurements in those tables were taken on a dual processor Intel Xeon E5645 2.4 GHz with six cores each and 48 GB of memory for the CPU version and a NVidia GeForce GTX 580 with 512 cores, 1.5 GB of memory and a memory bandwidth of 192.4 GB/s for the GPU version. The compiler used was gcc (GNU), version 4.4.7, and the operating system was Linux Ubuntu.

For a specific example, explicit and implicit solvers cannot be directly compared in terms of execution time, because the time discretization of the explicit solver is more restrictive than the one of the implicit solver. The scale factor of *η* = 100 for all implicit cases was arbitrarily chosen but with the restriction of having enough resolution in the temporal discretization (stability was actually observed for *η* > 100).

The myelinated axon represents the perfect scenario to exploit the parallel versions of *Neurite*. With a considerable number of elements (∼ 251,000) the GPU implementation of the program is much faster than the sequential implementation, reducing the execution time from days to minutes (see Table 3). This performance is justified by the parallel structure of the GPUs (initially aimed at accelerating image processing), for which large amount of data, stored in matrices, are managed inside the graphics cards. Although the GPU implementation is always much faster than the CPU version, the speedup (i.e., how much faster the parallel implementation is compared to the CPU implementation) for the explicit scheme is sensibly smaller than for the implicit scheme (see Table 3). This is due to the different parallel approaches used for each solver.

**Fig 7:**
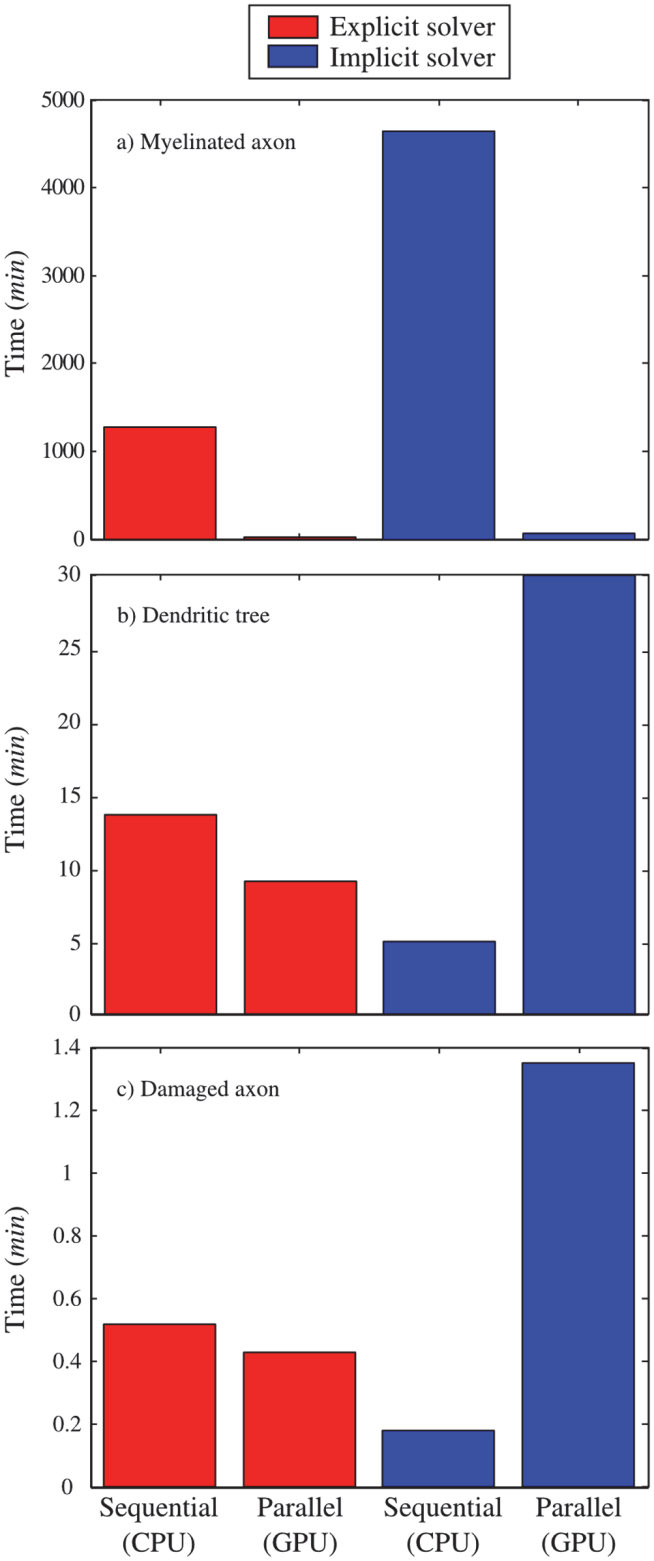
Performance of the solvers and processors. a) The myelinated axon is the ideal scenario to exploit the GPU implementation of *Neurite*, where the time consumptions is reduced from days to minutes. For the dendritic tree b) and the damaged axon c), the GPU implementation did not show any advantage compared to the CPU implementation.

The results are graphically shown in Fig. 7. The GPU implementation is slower than the CPU version when the number of elements is not large enough to have all threads of the GPU in the graphic card working at the same time: thus indicating that one should consider the CPU implementation of the explicit and implicit solvers for small examples, see Tables 4 and 5. This behavior of the GPU version was predictable, as it is mainly designed to simulate efficiently large scenarios.

## Discussion

A new simulator coupling mechanical and electrophysiological properties in neurites was presented here. *Neurite* is a versatile program that simulates the electrical signal propagation in neurites under mechanical loading, with sequential CPU and parallel GPU versions. The flexibility of the program was shown with three different applications: long myelinated axon, dendritic tree and axon under mechanical loading. The observed excellent performance of the GPU parallel implementation of the solvers opens the door to very large scale simulations.

When restricted to its electrophysiological components, *Neurite* can directly be compared to other simulators [13, 15, 16, 19, 24, 27]. Although this computational approach of the electrophysiological part of *Neurite* is not new, the way of solving the PDEs inside *Neurite* provides the necessary versatility to be coupled, extended, or adapted to different aims. More specifically, the FDM provides the mathematical simplicity and flexibility needed to implement new biological models and explore new parallel techniques such as MICs or GPUs. The main novelties of *Neurite* are its ability to simulate the electrical signal propagation under mechanical loading and the high performance achieved by the parallel version, implemented using GPUs. It must also be emphasized that the convergence of the finite difference scheme was systematically checked by use of spectral analysis, whereas, for some of the references mentioned above, the convergence was explored at best by halving the spatial and/or the temporal discretizations (for consistency) and empirically keeping a small time step (for stability), but in general, convergence was not fully studied or at least not demonstrated. Additionally, as can be inferred from the Rallpacks validation, *Neurite* is faster than the compartmental models (i.e., NEURON [23] and GENESIS [52]) as long as the element size is large enough (see S1 File and Ref. [44] for more details).

In the first application example, the GPU version of *Neurite* shows a high performance for a large number of elements. Two examples of parallel computing with neuronal models are the well established simulation environments NEURON [23] and GENESIS [52]. The parallel implementations of both programs focus on high performance computing by means of multiprocessors such as workstations, small clusters, or supercomputers (Ref. [53, 54] for NEURON and Ref. [55] for GENESIS). These approaches require a multiprocessor computer with a considerable number of processors in order to reach a good speedup. Only recently, Ben-Shalom and coworkers have implemented an accelerating compartmental algorithm in NEURON with GPU coding adaptations, allowing for simulations 150-fold faster than the CPU versions [56]. GPU approaches thus appear as a very good environment to exploit parallel simulations for large scale modeling [57–60]. Additionally, the number of accelerator-based supercomputers in *top500* (www.top500.org) shows a clear trend in the adoption of this technology towards the exascale simulation horizon. The second application demonstrates the ability of *Neurite* to work with segmented neurons and a third-party database, whereas the third application exhibits the ability of *Neurite* to simulate the electrical signal propagation under mechanical loading [11].

*Neurite* envisions many different future applications. The soma can be easily added as another element in the discretization with its corresponding geometrical and electrical properties (e.g., a sphere with the corresponding ion channels population). With this improvement, *Neurite* will be able to simulate a whole neuron. Other alternatives to HH are also easily implementable as a new subclass of the discretization class, or by adapting properly the properties of the ion channels (e.g., rate constant equations, dimensionless activation/inactivation particles, conductances) [61], at least in the CPU version of the program. Future implementations of synaptic models simulated by means of Monte Carlo techniques or PDEs (see Ref. [62]) will then allow for small networks, thus leveraging the promising performance of the GPU implementation. Finally, the program will be improved to simulate compound action potentials instead of APs, by averaging the potential based on the corresponding experimental methods used for the measurements in nerves.

Implementation-wise, the use of different architectures to further improve the performance of the parallel version, i.e., multicore processors and MICs, is ongoing. Indeed, although the GPUs have been chosen, the new MIC architecture with its 60 cores, 4-way SMT (Simultaneous multithreading) per core and 512-bit vectorial units (SIMD) appears as another excellent candidate. As these same characteristics are also exploited in the GPUs, a good performance can a priori be expected for this architecture. Multicore computers could also be used with OpenMP programming to provide a good performance even when coprocessors are not available in the computer.

## Supporting Information

S1 File *Neurite* validation against Rallpacks. **Neurite**: available under academic license on http://senselab.med.yale.edu/ModelDB/ShowModel.asp?model=168861.
(DOC)
