# Supplementary material for: Neurite, a Finite Difference Large Scale Parallel Program for the Simulation of Electrical Signal Propagation in Neurites under Mechanical Loading
Source: PLoS One. 2015 Feb 13;10(2):e0116532. doi: 10.1371/journal.pone.0116532 (PMC4334526; doi:10.1371/journal.pone.0116532)
Supplement: S1 File — Neurite: available under academic license on http://senselab.med.yale.edu/ModelDB/ShowModel.asp?model=168861. (DOC) [file pone.0116532.s001.doc]

***Neurite*, a finite difference large scale parallel program for the simulation of electrical signal propagation in neurites under mechanical loading**

Julián A. García-Grajales, Gabriel Rucabado, Antonio García-Dopico, José-María Peña, Antoine Jérusalem

**1 IMDEA Materials Institute, Getafe, Madrid, Spain**

**2 DATSI Computer Science, Universidad Politécnica de Madrid, Madrid, Spain**

**3 Madrid Supercomputing and Visualization Center**

**4 Department of Engineering Science, University of Oxford, Oxford, UK**

**5 Mathematical Institute, University of Oxford, Oxford, UK**

**Corresponding author: antoine.jerusalem@eng.ox.ac.uk**

## Supporting Information 1

### *Neurite* validation

The validation of *Neurite* against the Rallpacks benchmarks [1] is provided here. To this end, the program is run for all three scenarios: a passive sealed-end cable (Rallpack 1), a passive dendritic tree (Rallpack 2) and an unmyelinated axon (Rallpack 3). In all cases, the current is applied at the beginning of the neurite, and the evolution of the potential is recorded at this point as well as at the tip of the unbranched neurites, or at the last tip for the branched cable. See Ref. [1] for more information about the electrical properties and final configuration of each scenario. Note that NEURON [2] and GENESIS [3] are making use of compartmental models whereas *Neurite* uses FDM.

The results are shown in Tables 1, 2, 3 and 4. The simulation speed and the accuracy (compared to the analytical solutions for Rallpacks 1 and 2, and to NEURON and GENESIS for Rallpack 3) are presented. Note that “Simulation speed” and “Accuracy” refer to metrics proposed by Rallpacks, see Ref. [1] for more details. *Neurite* performed adequately for all tests suggested by the Rallpacks benchmarks for the implicit version of the program. For the explicit scheme, the time step is conditioned by the critical time step, and the Rallpacks studies are not applicable.

**Table 1.** **Rallpack 1. Passive cable**

| $\Delta t(\mu s)$ | Simulation speed | Accuracy |
| --- | --- | --- |
| 2 | 0.38 | 0.03 |
| 20 | 1.29 | 0.86 |
| 200 | 4.39 | 4.39 |

The number of elements is $1,$ $002$. Note that the number of elements is not $1,$ $000$ as suggested by the corresponding benchmark because of the way the sealed-end boundary conditions are applied in *Neurite*.

**Table 2.** **Rallpack 2. Passive dendritic tree**

| $\Delta t(\mu s)$ | Simulation speed | Accuracy |
| --- | --- | --- |
| 128 | 128 | 0.05 |
| 1280 | 384 | 0.33 |
| 12800 | 384 | 3.34 |

The number of elements is $1,$ $536$. Note that the number of elements is not $1,$ $000$ as suggested by the corresponding benchmark because of the way the sealed-end boundary conditions are applied in *Neurite*. It is remarkable that if the element size is larger, *Neurite* performs much faster than NEURON and GENESIS, see Ref. [1] for a better comparison.

**Table 3.** **Rallpack 3. Unmyelinated axon**

| $\Delta t(\mu s)$ | Simulation speed | Accuracy vs. NEURON | Accuracy vs. GENESIS |
| --- | --- | --- | --- |
| 2 | 0.44 | 1.01 | 1 |
| 20 | 1.5 | 1.94 | 1.99 |
| 200 | 3.34 | 3.33 | 3.19 |

The number of elements is $1,$ $002$. Note that the number of elements is not $1,$ $000$ as suggested by the corresponding benchmark because of the way the sealed-end boundary conditions are applied in *Neurite*. The comparison is done against NEURON and GENESIS as the analytical solution of the equation is unknown.

**Table 4.** **Rallpacks. Explicit scheme**

| Rallpack | $\Delta t(ns)$ | Simulation speed | Accuracy |
| --- | --- | --- | --- |
| 1 | 8 | 0.23 | 0.02 |
| 2 | 500 | 16 | 0.02 |
| 3 | 18 | 0.062 | NEURON 0.83; GENESIS 0.89 |

The parameters for these simulations are the same as for the implicit scheme. The explicit scheme is slow for Rallpacks 1 and 3 and faster for Rallpack 2 (as a consequence of the element sizes used).

**References**

1. Bhalla U, Bilitch D, Bower J (1992) Rallpacks: a set of benchmarks for neuronal simulators. TINS 15: 453–548.

2. Hines M, Carnevale N (1997) The NEURON simulation environment. Neural Computation 9: 1179–1209.

3. Bower J, Beeman D (1998) The book of GENESIS: exploring realistic neural models with the GEneral Neural Simulation System New York. Springer-Verlag.
